# Patient reported outcome data from acromegaly patients treated with injectable somatostatin receptor ligands (SRLs) in routine clinical practice

**DOI:** 10.1186/s12902-020-00595-4

**Published:** 2020-07-31

**Authors:** Eliza B. Geer, Jill Sisco, Daphne T. Adelman, William H. Ludlam, Asi Haviv, Shuqian Liu, Susan D. Mathias, Dana Gelbaum, Lizheng Shi

**Affiliations:** 1 Memorial Sloan Kettering, New York, NY USA; 2 Acromegaly Community, Grove, OK USA; 3 Northwestern University, Chicago, IL USA; 4 Chiasma, Inc, Waltham, MA USA; 5 Tulane University, New Orleans, LA USA; 6 Health Outcomes Solutions, P.O. Box 2343, Winter Park, FL 32790 USA

**Keywords:** Acromegaly, Somatostatin receptor ligands, Treatment satisfaction, Patient reported outcomes, Questionnaire

## Abstract

**Background:**

Acromegaly patients managed on Somatostatin receptor ligands (SRLs), the most common first-line pharmacotherapy for acromegaly, may still experience acromegaly symptoms such as headache, sweating, fatigue, soft tissue swelling, and joint pain, even those with normal IGF-1. Additionally, treatment with SRLs may cause injection site reactions and other side effects such as gastro-intestinal (GI) symptoms. This study utilized patient-reported outcome measures to examine the burden associated with acromegaly and its treatment for patients receiving a stable dose of long-acting SRLs in routine clinical practice.

**Methods:**

US acromegaly patients on a stable dose of SRL seen by their treating healthcare provider in the past 12 months completed a one-time online survey including the Acro-TSQ, an acromegaly-specific tool for assessing symptom burden and treatment satisfaction and convenience.

**Results:**

One hundred five patients were enrolled (mean age 49.9 years, 79.1% female). Patients experienced numerous symptoms, including > 80% who experienced joint pain, acro-fog, swelling of soft tissue, and fatigue/weakness. Many symptoms occurred constantly, while some occurred at the end of the injection cycle, even among those with IGF-1 < = 1.0 ULN. Injection site reactions were common. Patients were moderately satisfied with their current treatment; symptoms and side effects often affected daily activities. On average, patients reported > 3 acromegaly provider visits/year.

**Conclusions:**

Despite receiving a stable dose of SRL and regular visits with an acromegaly healthcare provider, US acromegaly patients in routine clinical practice, and even the subgroup with normal IGF-1, report significant burden of disease and treatment.

## Background

Acromegaly is an uncommon hormonal disorder most often due to a pituitary gland tumor. Overproduction of growth hormone (GH) and insulin-like growth factor (IGF-1) may result in changes to facial appearance and enlargement of the hands and feet. In addition other signs and symptoms are common [1–4]. Somatostatin receptor ligands (SRLs) are the most common first-line medical therapy [5, 6] and are administered as either intramuscular (octreotide) or deep subcutaneous injections (lanreotide). Current treatment options for acromegaly come with limitations: potential side effects associated with SRLs include injection site reactions and gastro-intestinal (GI) symptoms [7, 8], and patients managed on SRLs, even those with normal IGF-1, may still experience acromegaly symptoms that interfere with daily life, leisure, and work [9].

There is a growing appreciation for the importance of patient-centered care [10]. Patient-reported outcome (PRO) measures are considered by many to be as important as clinical or physiological outcomes, and there exist several sources, including published texts and guidelines from the Institute of Medicine and the Food and Drug Administration for how to effectively develop these types of measures and use them in clinical trials, as well as how they may be used to document disease burden and guide interventions and disease management [11–13].

The burden of acromegaly and its treatment in the US and European Union has been reported elsewhere, including its economic burden and impact on health-related quality of life [9, 14, 15]. This study used patient-reported outcome measures to further understand the disease and treatment burden for US acromegaly patients treated with long-acting SRLs in routine clinical practice.

## Methods

### Study type and patient population

Acromegaly patients included in this cross-sectional, US-based study (NCT# 03613623) were recruited primarily through the Acromegaly Community, Inc. (www.acromegalycommunity.org) and by clinical practices in the US in 2018. Acromegaly is a rare condition, so enrollment estimates were pragmatic and not based on power calculations. The goal was to enroll at least 100 patients. To be eligible, adults (aged 18 to < 95 years) reported a diagnosis of acromegaly which was subsequently confirmed by the patient taking a knowledge screening questionnaire that required them to recall their current medical treatment and doses. Regardless of how they were identified, interested participants were directed to the REDCap system maintained by Tulane University and were screened for eligibility.

To be eligible for the study, patients needed to be currently treated with first generation injectable SRLs including octreotide or lanreotide for ≥12 months with no adjustment in dose during or following their most recent acromegaly health care provider (HCP) office visit (i.e., those with stable disease), and were required to have been evaluated by their treating acromegaly HCP within the past12 months (plus or minus 2 months). Finally, patients were also required to be able to read and understand English, to reside in and undergo acromegaly treatment in the US, and be willing and able to sign the informed consent. Patients were excluded if they had participated in an octreotide capsules trial or were currently treated with pegvisomant monotherapy or pasireotide. This study was approved by the IRB at Tulane University (IRB #2018–879), and enrolled patients provided informed consent to participate via electronic signature.

Participating patients completed an online survey containing questions developed specifically for this study and a newly developed measure, the Acro-TSQ. The survey asked about characteristics and management of their acromegaly, frequency severity, and timing of symptoms, level of biochemical and symptom control, adverse treatment reactions, and overall health. Sample items from the survey are included in a supplemental file. The online survey also included the Acro-TSQ, an acromegaly-specific PRO assessing symptom and GI side effect interference, treatment satisfaction, treatment bother, and treatment convenience [16, 17]. The Acro-TSQ contains 24 items and was developed in line with recommendations by the Food and Drug Administration in a published document containing guidelines for PRO development which included qualitative research with individuals with acromegaly [12]. Those interested in its use should contact WHL. Patients’ HCPs were contacted and provided the patient’s most recent IGF-1 value available. Discordance between outcomes reported by acromegaly patients treated with long-acting SRLs and those perceived by their HCP have been reported elsewhere [18].

### Statistical analysis

Descriptive analyses of patient data included frequencies and percentages or means, standard deviations (SDs), and ranges on demographic (age, gender) and clinical characteristics (duration of disease, current treatment, history of other therapies), routine management, active symptoms, adverse drug reactions, general health rating, and treatment satisfaction. Descriptive analyses were also performed on Acro-TSQ domain scores (symptom interference, GI side effect interference, treatment satisfaction, injection site interference, emotional reaction, and treatment convenience), which can range from 0 (most symptomatic/interference) to 100 (least symptomatic/interference). Subgroup analyses were performed on select survey responses and Acro-TSQ domain scores by patient characteristics such as gender, the number of symptoms, IGF-1 level, and the level of symptom control using t-tests or the Wilcoxon test for continuous variables and chi-square or Fisher’s exact test for categorical variables. All results were based on patient self-report.

## Results

### Demographic and clinical characteristics

A total of 112 of 146 eligible patients (response rate of 77%) were identified and signed consent forms. Of those, 105 (94%) completed the online survey and are included in the analysis. The mean (SD) age for these 105 patients was 50 (12.5) years, 79% were female, and the mean (SD) duration of disease was 10 (8.1) years (Table 1). The distribution of current SRL treatment included 42 (40%) on octreotide and 63 (60%) on lanreotide. Among patients receiving octreotide, 67% were on “low” or “middle” level doses (< 30 mg total/month); 62% of patients receiving lanreotide were on “low” or “middle” level doses (< 120 mg total/month). It was common for acromegaly to be treated solely with SRL therapy (63% of patients). However, some patients received multiple medications for acromegaly in addition to an SRL, 17% received combination therapy with pegvisomant, 11% with cabergoline, and 5% with both pegvisomant and cabergoline.

**Table 1:** Patient Self-Reported Demographic and Clinical Characteristics

**Characteristic**	**Results**
Total Number	105
Female, % (N)	79% (83)
Age, Years, Mean ± SD	50 ± 12.5
Duration of Acromegaly, Years, Mean ± SD	10 ± 8.1
Current SRL, % (N)	
Octreotide (*n* = 42)	40% (42)
Low dose (< 20 mg total/month)	29% (12)
Middle dose (20 mg to < 30 mg total/month)	38% (16)
High dose (≥ 30 mg total/month)	33% (14)
Lanreotide (*n* = 63)	60% (63)
Low dose (< 90 mg total/month)	24% (15)
Middle dose (90 mg to < 120 mg total/month)	38% (24)
High dose (≥120 mg total/month)	38% (24)
Procedure, % (N)	
Pituitary surgery only	61% (64)
Both pituitary surgery and radiotherapy	30% (31)
Neither pituitary surgery or radiotherapy	10% (10)
Time Since Last Surgery, Years, Mean ± SD (*n* = 95)	9 ± 7.3
Time Since Last Radiotherapy, Years, Mean ± SD (*n* = 31)	9 ± 7.9
Medications for Acromegaly, % (N)	
SRL Only	63% (66)
SRL + Pegvisomant	17% (18)
SRL + Cabergoline	11% (11)
SRL + Pegvisomant + Cabergoline	5% (5)
Unknown	4% (4)
Self-perception of symptom control, % (N)	
Well controlled	29% (30)
Partially controlled	48% (50)
Not controlled	22% (22)
Not sure	3% (3)
IGF-1, ULN, Mean ± SD (*n* = 47)	0.85 ± 0.56
IGF-1 < = 1 ULN	79% (37)
IGF-1 > 1 ULN	21% (10)
Self-perception of biochemical control	
Well controlled	66% (69)
Partially controlled	26% (27)
Not controlled	5% (5)
Not sure	4% (4)

*IGF-1* Insulin-like growth factor 1, *SRL* Somatostatin receptor ligand, *SD* Standard deviation, *ULN* Upper limit of normal

### Injections and routine disease management

According to patient responses, 56% received their injection at home, 33% at a local doctor’s office, 8% at an outpatient hospital, and 2% at a regional or community clinic (data not shown). Roughly half (48%) reported that they self-injected or received injections from their spouse or informal caregiver. The remainder received their injection by their doctor (7%) or nurse or other healthcare professional (48%). The mean number of reported visits to see an acromegaly provider was 3.12 per year (median = 2, range = 1 to 13). More than half of patients (53%) indicated they had 1 to 2 visits per year; 39% responded that they had between 3 and 5 visits per year, and 8% indicated they had 6 or more visits in a year. Eighty-four percent reported seeing their provider in the previous 6 months.

### Symptoms, control, and injection-site reactions

IGF-1 data were reported by each patient’s HCP and not directly by each patient. Not all HCPs agreed to provide this information; results were available for 47 patients (45%). The mean (SD) IGF-1 was 0.85 (0.56), including 37 (79%) with IGF-1 < = 1 ULN and 10 patients (21%) with IGF-1 > 1 ULN.

Patients rated their self-perception of biochemical control as well-controlled [69 (66%)], 32 (31%) responded that they were not well-controlled (including 27 [26%] who indicated that they were “partially controlled” and 5 [5%] who said they were “not controlled”), and 4 patients or 4% indicated that they were “not sure” (Table 1).

When asked about symptom control, 30 (29%) of patients responded that their symptoms were well-controlled, 72 (69%) said their symptoms were not well-controlled (including 50 [48%] who said they were “partially controlled,” and 22 [22%] who said they were “not controlled.”) Three patients (3%) were “not sure” of their level of symptom control.

**Fig. 1:**
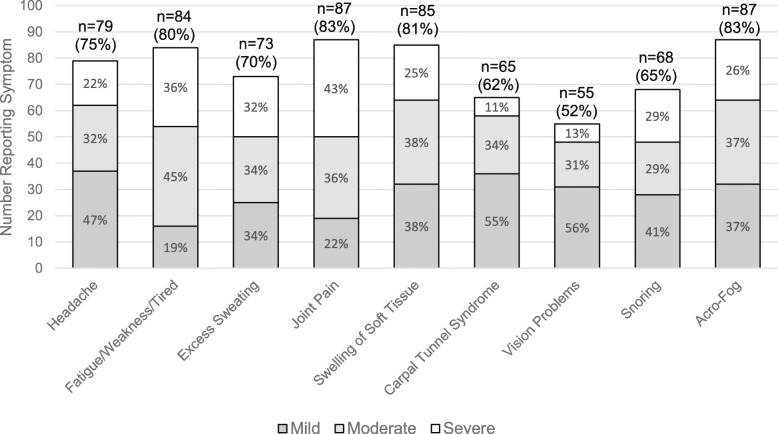
Frequency and Severity of Reported Symptoms. The height of a stacked bar reflects the number who reported experiencing that symptom (total count is presented above bar and the percent it represents out of the total sample of 105 is in parentheses); Percent shown inside of each bar section is out of those who experienced that symptom

**Fig. 2:**
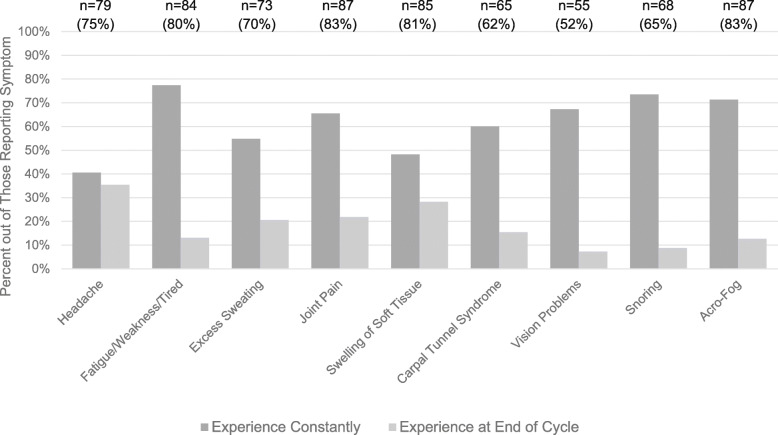
Pattern of Symptom Occurrence. Total number and percent who reported experiencing the symptom out of the total sample of 105 is presented at the top of the graph. Heights of individual bars reflect the percent of those who experienced that symptom who indicated that they occurred constantly or at the end of the cycle

A variety of symptoms was reported by the majority of respondents: 4 symptoms were reported by 80% or more of patients. The most frequently reported symptoms included joint pain and acro-fog (a short-term memory loss or feeling in a daze) (83% for both) followed by swelling of soft tissue (81%), fatigue/weakness/tired (80%), and headache (75%) (Fig. 1). Patients often reported experiencing symptoms constantly, including 77% of those reporting fatigue, weakness or tiredness, 74% of those reporting snoring, 71% of those with acro-fog, 67% of those with vision problems, 66% of those with joint pain, 60% of those with carpal tunnel, 55% of those with excess sweating, 48% of those with swelling of soft tissue, and 41% of those with headache (Fig. 2). However, headaches, swelling of soft tissue, joint pain and excess sweating were also common at the end of the injection cycle (> 20%).

A similar pattern was observed among the subgroup whose IGF-1 < = 1 ULN (*N* = 37). The most frequently-cited symptoms were joint pain (76%), swelling of soft tissue (76%), and acro-fog (76%), followed by fatigue, weakness, or tiredness (73%) and headache (70%, Table 2). Symptoms most commonly reported as being severe within this subgroup included acro-fog (32% of those reporting the symptom indicated it was “severe”), joint pain (32%), headache (31%), fatigue, weakness, tiredness (30%), and swelling of soft tissue (29%). Symptoms of acro-fog (89%), fatigue, weakness, or tiredness (82%), snoring (68%), carpal tunnel syndrome (67%), joint pain (64%), and vision problems (61%, Table 2) occurred constantly. The most common symptoms reported occurring at the end of the cycle were swelling of soft tissue (36%), headache (35%), joint pain (29%), and carpal tunnel syndrome (24%, Table 2).

**Table 2:** Frequency, Severity, and Pattern of Symptoms for Patients Whose IGF-1 < = 1 ULN; *N* = 37

**Symptom**	**Experienced Symptom Yes % (N)**	**Reported Severity**			**Pattern of Symptom Occurrence**				
		**Mild %**^**a**^**(n)**	**Moderate %**^**a**^**(n)**	**Severe %**^**a**^**(n)**	**Constant %**^**a**^**(n)**	**Right After Injection %**^**a**^**(n)**	**Middle of Cycle %**^**a**^**(n)**	**End of Cycle %**^**a**^**(n)**	**Not sure %**^**a**^**(n)**
Headache	70% (26)	42% (11)	27% (7)	31% (8)	46% (12)	8% (2)	0% (0)	35% (9)	12% (3)
Fatigue/ weakness/ feeling tired	73% (27)	22% (6)	48% (13)	30% (8)	82% (22)	0% (0)	4% (1)	7% (2)	7% (2)
Excess sweating	57% (21)	52% (11)	33% (7)	14% (3)	43% (9)	10% (2)	5% (1)	19% (4)	24% (5)
Joint pain	76% (28)	25% (7)	43% (12)	32% (9)	64% (18)	0% (0)	0% (0)	29% (8)	7% (2)
Swelling of soft tissue	76% (28)	36% (10)	36% (10)	29% (8)	43% (12)	7% (2)	4% (1)	36% (10)	11% (3)
Carpal tunnel syndrome	57% (21)	71% (15)	19% (4)	10% (2)	67% (14)	0% (0)	0% (0)	24% (5)	10% (2)
Vision problem	49% (18)	61% (11)	22% (4)	17% (3)	61% (11)	0% (0)	6% (1)	6% (1)	28% (5)
Snore	51% (19)	53% (10)	32% (6)	16% (3)	68% (13)	5% (1)	0% (0)	11% (2)	16% (3)
Acro-fog	76% (28)	18% (5)	50% (14)	32% (9)	89% (25)	0% (0)	0% (0)	7% (2)	4% (1)

^a^Of those who reported experiencing the symptom

Among all patients, common injection site reactions included pain during the injection (83%) or pain for several hours (68%) or days (49%) afterwards, and nodules (63%). Swelling (47%), bruising (45%), and scar tissue/hardness of skin (42%) were also reported (Table 3).

**Table 3:** Frequency and Severity of Injection Site Reactions, All Patients

**Injection Site Reaction**	**Experienced % Yes (N**^**a**^**)**	**Mild % (n**^**b**^**)**	**Moderate % (n**^**b**^**)**	**Severe % (n**^**b**^**)**
Pain at injection site **during** injection	83% (87)	56% (49)	31% (27)	13% (11)
Pain at injection site **several hours** after injection	68% (71)	60% (43)	32% (23)	7% (5)
Pain at injection site **several days** after injection	49% (51)	65% (33)	28% (14)	8% (4)
Bruising at the injection site(s)	45% (47)	70% (33)	30% (14)	0% (0)
Swelling at the injection site(s)	47% (49)	65% (32)	33% (16)	2% (1)
Nodules (knots and bumps under the skin) at the injection site(s)	63% (66)	47% (31)	42% (28)	11% (7)
Scar tissue/hardness of the skin at the injection site(s)	42% (44)	41% (18)	46% (20)	14% (6)

^a^Of total sample

^b^Of those who reported experiencing the injection site reaction

### Acro-TSQ results

The mean domain scores (possible range of 0 to 100) in order from lowest (most interference) to highest (least interference) were symptom interference (51.4), treatment satisfaction (53.9), treatment convenience (62.9), emotional reaction (71.0), GI interference (71.4), and injection site interference (85.1).

Several statistically significant differences (*p* < 0.05) in domain scores were observed when stratified by patient characteristics (data not shown). For instance, mean scores for symptom interference, GI interference, and treatment satisfaction were lower (worse) for those who self-reported a higher number of symptoms (of any severity as well as by the number of moderate and severe symptoms). Additionally, mean scores for symptom interference and treatment satisfaction were significantly lower for those who self-reported that their disease was not well controlled versus those who considered their disease to be well controlled. However, there were no significant differences in domain scores when evaluated by IGF-1 values, by gender, age group (<= 50 years vs 50 years and older), duration of disease, drug regimen, or practice setting.

### Acro-TSQ item-level results

When asked about their current treatment for acromegaly, 39% (41) rated their current treatment as “very good” or “excellent,” and 37% felt their current treatment is “convenient” or “somewhat convenient;” 17% (18) were “very satisfied,” 19% (20) were “satisfied,” and 23% (24) were “somewhat satisfied” with their current treatment. The percent of patients who indicated that they experience acromegaly symptoms despite receiving treatment was 87%, which is larger than the percent who reported that they felt their symptoms were not well-controlled (69%). Additionally, 72% (76 of 105) experienced GI side effects, lasting a mean (SD) of 10 (9.8) days post injection; 84 and 86% indicated that GI side effects interfered with daily activities and leisure activities, respectively. Patients were bothered by several aspects of treatment, including: the amount of time they experienced symptoms (100%; 91 of 91), injection site reactions during the first few days (74%; 40 of 54), the need to schedule injections (65%; 62 of 95), and having to travel for injections (74%; 43 of 58). The mean (SD) self-reported general health rating was 61 (20.7), with ratings ranging from 18 to 100.

## Discussion

Acromegaly patients in this study report a variety of symptoms. Several symptoms were common: among all patients, four symptoms were each experienced by more than 80% of patients. Numerous symptoms were experienced constantly by a majority of respondents; a minority of patients experienced certain symptoms at the end of the injection cycle. Notably, the subgroup of patients with IGF-1 < = 1 ULN also frequently experienced numerous symptoms that were commonly reported as occurring constantly. Five different symptoms (headache, fatigue, joint pain, swelling, acro-fog) were each experienced by at least 70% of these patients. Surprisingly, while 66% of the patients reported that they perceived their disease to be biochemically controlled, only 29% reported their symptoms were well controlled.

The percent of patients experiencing symptoms constantly (up to 77%) is notable considering that all patients were receiving treatment and 79% had IGF-1 < = 1 ULN. Additionally, about one-third of the patients reported symptoms worsening towards the end of an injection cycle. While these results are similar to those found in a previous study finding, [9] they remain surprising, since they reflect increases in pain and discomfort each cycle for many patients. The current study is the first to examine the pattern of symptom occurrence during the treatment cycle among US acromegaly patients.

Based on responses to the Acro-TSQ, 87% of patients continue to experience acromegaly symptoms that interfere with daily activities despite receiving treatment. The mean score for the treatment satisfaction domain was 54, suggesting that satisfaction with current treatment was moderate. GI side effects were experienced by 72% of patients, and frequently interfered with activities. In addition, subjects experienced pain, nodules, swelling, and bruising at the injection site. Those with symptoms did not always report any associated significant bother. That these patients experience symptoms of acromegaly and endure negative impacts of treatment despite seeing an acromegaly healthcare provider more than 3 times per year, on average, suggests that these patients experience significant burden of treatment and that there is unmet need in this population.

Previous studies have demonstrated that symptom burden and treatment can impact quality of life. Liu, et al. examined 106 patients from the Acromegaly Community in the US; 91% reported ongoing symptoms of acromegaly, and those with 4+ symptoms had lower health-related quality of life scores than those with 3 or fewer symptoms [19]. Treatment with SRLs has also previously been associated with reduced quality of life. In a study of 108 patients in the Netherlands who underwent pituitary surgery, patients who received an SRL postoperatively (due to persistent or recurrent disease) reported worse scores on physical functioning, fatigue, activity, vitality, and general health perception than those not receiving an SRL, even when the comparison was limited to patients with similar IGF-1 levels [20].

Previous studies have also observed that acromegaly patients treated with an SRL frequently experience acromegaly symptoms and treatment side effects. In a study of 195 patients in Germany, the UK, and the Netherlands, Strasburger et al. [9] reported that 36% of those receiving long acting SRL injections (either octreotide or lanreotide) were biochemically controlled. More than 70% reported symptoms despite receiving treatment, with 52% reporting that symptom burden worsens towards the latter part of their monthly injection cycle, and 62% reporting that symptoms interfered with daily life. Further, 70% reported pain at the injection site; other injection-site reactions reported include nodules (38%), swelling (28%), bruising (16%), scar tissue (8%), and inflammation (7%). Despite these results, patients were generally satisfied with their current treatment, as was true in the current study.

The results of the current study are from a large, US-based sample of patients in usual care and are based on a comprehensive online survey which included a novel acromegaly-specific PRO measure. Patient recruitment resulted in a heterogeneous sample in regards to age, gender, and treatment (type of SRL, monthly dose, use of concomitant medications to treat acromegaly, prior pituitary surgery). Additionally, a wide variety of symptoms, injection site reactions and side effects are represented in this analysis.

The results should be viewed in light of several potential limitations. First, since acromegaly is a rare condition and is also known to be associated with several other co-morbidities, it was not feasible to exclude patients with additional health conditions. In addition, all acromegaly patients included in this study were receiving a stable dose of injectable SRL and had seen their treating provider within the past year. Further studies are necessary to determine if the results observed herein are generalizable to other patient populations with acromegaly. Additionally, as most data were based on patient self-report, there may be some recall bias.

## Conclusions

This study illustrates that, even when US patients with acromegaly are receiving a steady regimen of first generation injectable SRLs and seeing their providers regularly, these patients in a real world setting report significant burden of disease including incomplete control of their symptoms that interferes with their daily life, leisure and work activities, often throughout the treatment cycle. Surprisingly, this remains true among those whose IGF-1 < = 1 ULN. This significant burden of disease and inadequate symptom control, particularly for individuals with IGF-1 < = 1 ULN, indicates an unmet need for patients with acromegaly. These findings highlight the importance of collecting and monitoring patient-reported outcomes for this population so that clinicians can incorporate these data to better manage their patients.

## Supplementary information

**Additional file 1.** Sample Items from the Online Survey.

## Data Availability

The datasets used and/or analyzed during the current study are maintained at Tulane University and are available from the corresponding author on reasonable request.

## Abbreviations

GH: Growth hormone
GI: Gastro-intestinal
HCP: Health care provider
IGF-1: Insulin-like growth factor
PRO: Patient-reported outcome
SRL: Somatostatin receptor ligands
UK: United Kingdom
US: United States
