# Supplementary material for: Patient reported outcome data from acromegaly patients treated with injectable somatostatin receptor ligands (SRLs) in routine clinical practice
Source: BMC Endocr Disord. 2020 Jul 31;20:117. doi: 10.1186/s12902-020-00595-4 (PMC7393879; doi:10.1186/s12902-020-00595-4)
Supplement: Supplementary file 1 — Additional file 1. Sample Items from the Online Survey. [file 12902_2020_595_MOESM1_ESM.docx]

**Supplementary File: Sample Items from the Online Survey**

**ACROMEGALY SYMPTOMS**

Please indicate which symptoms you typically experience. If you experience the symptom, please indicate the severity and the pattern of symptom over the course of the injection cycle.

Do you experience fatigue/weakness/feeling tired?

Yes /No

If Yes:

| Would you describe this as mild, moderate, or severe? | | | | Pattern of Acromegaly Symptom over the Injection Cycle  Please select the option that best describes pattern of acromegaly symptom (Check all that apply) | | | | |
| --- | --- | --- | --- | --- | --- | --- | --- | --- |
| Mild | Moderate | Severe | Not sure | Constant over entire injection cycle | Just after you receive the injection | At the middle of the injection cycle | At the end of the injection cycle before your next injection is due | Not sure |
|  |  |  |  |  |  |  |  |  |

**INJECTION-RELATED SIGNS AND SYMPTOMS (PHYSICAL IMPACT)**

Think specifically about the symptoms you have experienced that are related (or may be related) to any of your current injections. Please rate the severity for each symptom.

| **Injection Issues** |  |  |  |  |
| --- | --- | --- | --- | --- |
| Pain at the injection site: During the injection | None | Mild | Moderate | Severe |

**ACROMEGALY CONTROL**

In your opinion, how well are your acromegaly symptoms controlled?

Not controlled  Partially controlled  Well controlled  Not sure
